# Draft Genome Sequence of *Deinococcus* sp. Strain S9, Isolated from Microbial Mat Deposits of Hot Springs Located atop the Himalayan Ranges at Manikaran, India

**DOI:** 10.1128/MRA.00316-19

**Published:** 2019-07-11

**Authors:** Chandni Talwar, Amit Kumar Singh, Durgesh Narain Singh, Shekhar Nagar, Yogendra Singh, Mallikarjun Shakarad, Ram Krishan Negi, Rup Lal

**Affiliations:** a Department of Zoology, University of Delhi, Delhi, India; b Department of Biotechnology, Jamia Millia Islamia, New Delhi, India; c PhiXGen Private Limited, Gurugram, Haryana, India; University of Maryland School of Medicine

## Abstract

Here, we present the draft genome sequence of Deinococcus sp. strain S9, a red-pigmented and moderately thermophilic bacterium isolated from microbial mat deposits around the hot springs at Manikaran, Himachal Pradesh, India. The draft genome (3.34 Mb) contains 101 contigs with an average GC content of 66.4%.

## ANNOUNCEMENT

Our group has been incessantly exploring the microbial diversity at the geothermal hot springs located at Manikaran, Himachal Pradesh, India (1–7). Previously, we reported the draft genome sequence of Deinococcus sp. strain RL, which was isolated from sediments of this hot spring (6). In continuation of our efforts to study the extremophilic microbial diversity, we isolated a red-pigmented bacterium, Deinococcus sp. strain S9, from microbial mat deposits around the hot spring located at an altitude of 1,760 m at Manikaran (32°020′N, 72°210′E), Himachal Pradesh, India (surface temperatures, >57°C) (4). Briefly, 10 g of microbial mat sample was suspended in sterile normal saline solution and shaken at 45°C for 3 h. Serial dilutions from the seed sample were spread on Luria-Bertani agar (HiMedia, Mumbai, India) and incubated at 45°C. A red-pigmented bacterial colony optimally growing at 45°C was picked up after 24 h and designated S9. Based on 16S rRNA gene sequencing (1,278 bp), the isolate showed 99.06% identity with the type strain Deinococcus geothermalis DSM 11300.

The genomic DNA was isolated as described elsewhere (8). To determine the whole-genome sequence, library preparation was done using the TruSeq nano DNA PCR-free kit (catalog number FC-121-3001; Illumina, San Diego, CA), and sequencing was performed using the Illumina HiSeq 2500 platform. In total, 42,493,218 paired-end reads 100 bp long were generated and processed with Trimmomatic v0.39 (9) using default parameters. Assembly was performed using SPAdes v3.11.0 (10), by employing a multi-*k-*mer approach (*k* = 27, 31, 33, 35, 41, 45, 49, 53, 55, 75, 77, and 95), into 179 contigs. The assembly was validated by using QUAST v4.5 (11), and contigs <500 bp long were removed. The final assembly contains 101 contigs (*N*_50_, 111,497 bp) at 107-fold coverage. The total size of the draft genome is 3,344,786 bp, with an average GC content of 66.4%. The 16S, 23S, and 5S rRNA gene sequences (1 of each) were identified within the genome by using RNAmmer (12). In addition, 49 tRNA genes and 1 transfer-messenger RNA (tmRNA) gene were also predicted using ARAGORN (13). Nine CRISPR arrays along with several CRISPR-associated (*cas*) genes were also determined within the draft genome using CRISPRFinder (14).

Annotation using the NCBI Prokaryotic Genome Annotation Pipeline (PGAP) (15) predicted 3,108 protein-coding sequences. Enzymes involved in carotenoid biosynthesis, such as phytoene synthase, phytoene desaturase, and lycopene cyclase, were annotated within the draft genome (16, 17). The genomic studies of *Deinococcus* spp. are of great interest because of their high resistance to radiation, temperatures, and heavy metals and unparalleled efficiency in repairing damaged DNA (18). Several genes responsible for the DNA repair and resistance to radiation were also annotated, such as *uvrA* (19), *recO* (20), *recA* (21), *recF,* and *recR* (22). In addition, the gene coding for ClpX, which is involved in the repair of double-stranded breaks upon gamma irradiation (23), was also annotated. In-depth analysis of this genome would provide crucial details required for the understanding of the mechanisms involved in nucleotide excision repair, carotenoid biosynthesis, and survival at extreme niches.

### Data availability.

This whole-genome shotgun project has been deposited at DDBJ/ENA/GenBank under the accession number SKCF00000000 (BioProject number PRJNA525588 and BioSample number SAMN11053996). The version described in this paper is SKCF01 and consists of sequences SKCF01000001 to SKCF01000101. The raw reads are available in NCBI under BioProject accession number PRJNA525588.
